# Leader strategies for motivating innovation in individuals: a systematic review

**DOI:** 10.1186/s13731-020-00120-w

**Published:** 2020-05-12

**Authors:** Eleftherios K. Soleas

**Affiliations:** Faculty of Education, Queen’s University, Kingston, Canada

**Keywords:** Innovation, Expectancy–value theory, Systematic review, Leader approaches, Innovation education

## Abstract

Innovation is a topic of intense interest and is seen as key to confronting the vast majority of issues facing humanity. To consolidate the knowledge about approaches promoting innovation, this study conducted a systematic review integrating an all-database (*n* = 375) search through EBSCOhost completed on April 6th, 2019 in addition to search engine use. Three hundred three studies were full-text reviewed yielding 82 final studies eligible for the inclusion in findings extraction. The findings were synthesized and then organized into the Expectancy–value–cost (EVC) motivation framework to isolate promotive and hindering factors. It is clear that there is an unbalanced primacy in the innovation literature in favor of business and corporate settings with very little representation from the arts or social justice sectors. There is also a common trend of using surveys of individuals in organizations within a single discipline, while interviews are rare. The paucity of studying costs of innovation in the literature is symptomatic of the primarily positive psychology approach taken by studies, rather than a framework like EVC which also considers detractive factors like costs. Numerous studies provide support for the notion that more internal motivations like intrinsic (e.g., interest) and attainment (e.g., importance, fulfillment) were more influential than external motivators like rewards as targets of strategies. Leaders should focus, whenever possible, on topics that engaged curiosity, interest, and satisfaction and, if they choose to use rewards, should focus their strategies to give related rewards; otherwise, they risk sundering the internal motivation to innovate for already interested workers.

## Introduction

Innovation is key to confronting the vast majority of issues facing humanity. It can be defined variously and tends to include problem-solving processes (Cramond & Fairweather, 2013), executing novel ideas to create societal value (Soleas, 2018a) and applied creativity (Horkoff, Maiden, & Asboth, 2019). The capacity to refine existing ideas and challenge existing ideas is a very important and thankfully human process drawing intense interest from a range of disciplines including psychology (e.g., Carr, Kendal, & Flynn, 2016), business (e.g., De Clercq & Pereira, 2019), and education (e.g., Soleas, 2020), among many others. The importance, variety, and outcomes of innovation have made it necessary for institutions, corporations, and educational systems to more effectively stoke this crucial skill. It is the leaders, in these contexts, that must find strategies to motivate innovation. It follows that it should be a priority to understand what the leaders of various contexts can do to make innovation more likely.

This interdisciplinary perspective necessitates a broader view of the strategies which leaders (decision makers) can use to enact its promotion. Strategies will include the decisions, approaches, and interventions that the leading figures (leaders) in a given context, including teachers, managers, supervisors, and mentors can utilize to make innovation more likely. This review will consolidate the disparate literatures pertaining to the support of innovation in the varied disciplines through the use of strategies. It will identify lingering gaps in knowledge that would need to be explored to better inform innovation education efforts. As innovation is a multidisciplinary interest shared by business, economics, education, engineering, management, manufacturing, psychology, and many more disciplines, the resulting synthesis should be a shared understanding that reflects the diverse aspirations, goals, and perspectives of these disciplines (Baregheh et al., 2009; Soleas, 2020).

As will be revealed, there are some disciplinary studies of the motivations of innovation, with robust representation from business and economic disciplines (e.g., Manso, 2017; Marvel et al., 2007; Scott & Bruce, 1994). However, there are very few studies using a multidisciplinary perspective and none examining both promotive and hindering motivation factors for innovation from a multidisciplinary view. Although the interest is intense, the precise motivation factors underpinning this skill remain siloed in various disciplines. As a first step, it would be important to isolate and evaluate all the available evidence of the promotive and hindering motivating factors that would need to be addressed by those who seek to build learner capacity to innovate. This systematic review will bring together all available scholarly perspectives on the strategies available to leaders to motivate innovation with the goal of proving transferable strategies that can be adapted to a range of contexts.

## Motivating innovation

Expectancy–value–cost theory (EVC) is an established motivation framework that considers the factors that make motivation more likely and those that actively detract from motivation to complete a task (Barron & Hulleman, 2015; Flake et al., 2015). The promoting factors are grouped into expectancies (e.g., built expectations of success, perceived self-efficacy, and acquired confidence) and task values (interest, fulfilment, and utility). There are also factors that actively detract from motivation to innovate which are holistically termed “costs” which include prices that someone would pay to complete a task which can encompass pressure, stress, monetary considerations, and implications of failure.

Although EVC theory has recently begun to enjoy widespread popularity for explaining complex phenomena in the social sciences literature (e.g., Barron & Hulleman, 2015; Flake et al., 2015; Senko et al., 2011; Wigfield et al., 2009), it has been used very little in the study of innovation—and, surprisingly, not at all with respect to gaining insights into what approaches could motivate innovation, for instance, what leader or teaching strategies increase the confidence of learners for innovating. At the time of writing, the nebulous state of the literature remains siloed among different disciplines, and there has yet to be a cogent amalgamation of the research elucidating what motivates innovators in one place. What interventions can reduce the costs of innovating to the point where more learners are willing to give it a try? A systematic and two-pronged approach of evaluating the literature offerings on both promotive and hindering motivation factors promises a holistic view of the decision maker and teaching approaches that make innovation more likely and hindering factors that need to be addressed to make the most of promotion.

A typical literature review did not reasonably capture all the necessary information from such varied fields including business, education, and psychology, to name a few. Thus, a review that would be systematic, replicable, and thorough would be ideal to address this concern (Liberati et al., 2009; The Campbell Collaboration, 2017). Systematic reviews are structured literature reviews where researchers retrieve and evaluate all available evidence on a given topic; they synthesize, categorize, and appraise all the extant knowledge pertaining to a topic of inquiry. In this case, the review sought all articles through an EBSCOhost all-database search on approaches for promoting individuals’ capacity to innovate. As a result of innovation knowledge and, hence, promotion being spread across the many contributing disciplines, a siloing effect has occurred. To span these disciplines and evaluate all the knowledge currently available about approaches promoting innovation, a review that would be systematic, thorough, and replicable would be ideal to address this concern (Liberati et al., 2009; The Campbell Collaboration, 2017). A systematic review of the literature would provide the necessary insights to inform the design of approaches that would promote innovative behavior of aspirants.

## Methodology

For structuring the review, protocols as recommended by the Campbell Collaboration, a social sciences international collaboration that regulates and supports the conductance of rigorous systematic reviews of interventions in education and other settings were used (The Campbell Collaboration, 2017). In this case, this registered systematic review (osf.io/up83s) consolidated and analyzed empirical studies about the approaches for supporting innovation, which includes strategies, practices, interventions, and curricula, guided by the following research questions:

1. **What does the existing literature tell us about strategies that motivate and sustain human innovative behavior?**
  - What is found in the literature about strategies that build expectancies of individual innovative behavior?
  - What is found in the literature about strategies that build individual-held subjective task values of innovative behavior?
  - What is found in the literature about strategies that mitigate the perceived costs of individual innovative behavior?

A challenge with the current literature on innovation and a key reason why a systematic review is necessary is the nebulous and often conjectural ideas defining and explaining the motivation of innovators. To manage this issue, only empirical studies with human participants that examined the motivations of individuals as a unit of analysis were included. Additionally, only English-language articles and articles with verified English-language translations were considered. There were no restrictions on the years of included studies with the earliest studies dating back to 1967, and the most recent published in early 2019.

**Fig. 1:**
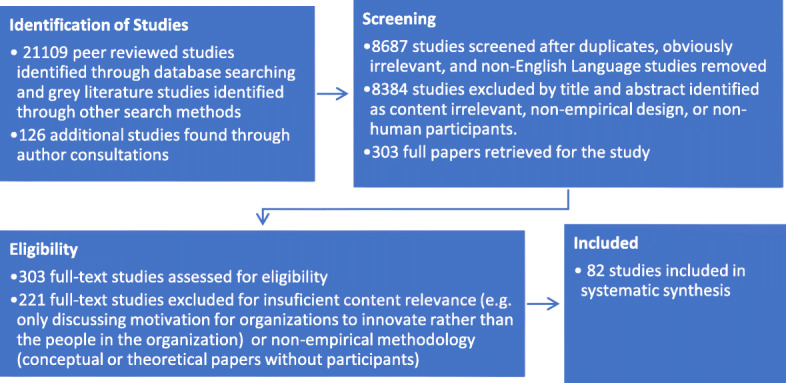
Flow of information in this systematic review study

Specific consideration was given to the design of strategies that build expectancies and values, and those that help mitigate the perceived and unperceived costs or risks of innovative behavior. This systematic review followed an all-database (*n* = 375) search through EBSCOhost performed on January 2nd, 2018 and again on April 6th, 2019, in addition to Google and Bing search engine use (see Fig. 1).

**Fig. 2:**
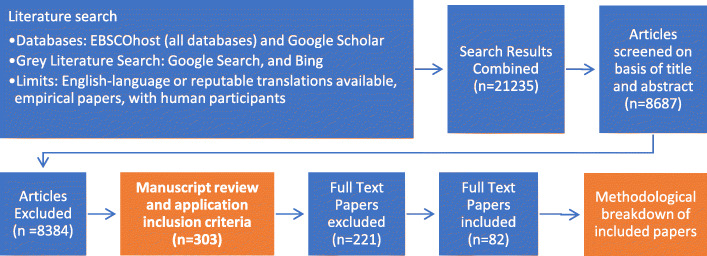
Flow diagram of the study selection

When contacting authors of included papers for additional sources to consider, there were no date-of-publication restrictions. One hundred twenty six additional studies from outside the database search were obtained this way, while an additional 85 studies were found to be already included in the search and removed as redundant. Database searching was concluded on April 6th, 2019, and the last of the article-yielding author replies was retrieved by February 1st, 2019 (see Fig. 2). The database searching was performed in early 2018, but subsequent searches updating with more recent articles were performed in April 2019 to include the most modern articles.

### Search strategy

In Abstract AND Paper: Innovat* AND (Motiv* OR Promot* OR Support*) AND (Strateg* OR Approach* or Interven*). Related word substitutions are allowed.

Using Boolean logic, the addition of an asterisk would tell the database to search for all permutations of the rest of the word. For example, motiv* would tell the database to search for motivation, motivator, motivate, motivating as related terms as well as synonyms like encourage, prompt, etc. As secondary search procedures, Google and Bing were also searched using an analogous process with Boolean search code operators. As well, email addresses of authors were extracted and these authors were contacted, resulting in 126 previously undiscovered prospective studies after eliminating redundant duplicates.

Research assistants were employed to facilitate the screening process by abstract and title. Study reviewers operated in dyads, with each member doing independent reviews of each abstract and title by deciding whether the title would be relevant and within the inclusion criteria. Each abstract and title was therefore reviewed twice for the purposes of adjudicating inclusion or exclusion. Disagreements were resolved through the study being included in the full paper review to avoid removing a potentially eligible study. Cohen’s Kappas were calculated using the tabulated data in the aggregate review (see Table 1). The Kappa value (0.806) indicates very good agreement among the reviewers. Three hundred three studies were full-text reviewed, of which 82 final studies were determined to be eligible for inclusion in findings extraction.

**Table 1:** Screening phase: review of titles and abstracts with relative agreement as indicated by Cohen’s Kappa

Aggregate			Kappa = 0.806	98.89% agreement
	Reject	Keep	SE of Kappa = 0.019	94.30% of the agreement could be explained by chance
Reject	8384	41	95% CI = 0.768 to 0.844	
Keep	55	207	Rated as very good	

Findings were extracted from the final papers using qualitative data analysis software, Atlas.ti v8.0. In this way, salient hypotheses, methodologies, findings, conclusions, participant data, and other articles for consideration were isolated from the full-text files. From the methodology, the paradigm (e.g., qualitative, quantitative, or mixed methodology) and tradition (e.g., case study, experimental design, or pre-/post-test) were isolated and extracted.

## Demographics of the literature results

Business workers and employees were the most common study participants (40.2%), followed by students (19.5%), business leaders (10.97%), consumers (10.97%), teachers (9.76%), and entrepreneurs (8.53%; see Table 2 for a complete listing of studies by participant type). Many studies examined the activities of managers or leaders as crucial for promoting the innovative behavior of other workers. For simplicity’s sake, the term leader will be used going forward for individuals who influence subordinate, worker, or learner groups in their care. In the case of schools, leaders would be those leading classes, namely teachers and administrators.

**Table 2:** Study participant type of the included studies

Business employees	Business leaders	Entrepreneurs	Teachers	Consumers	Students
1. Aalbers et al. (2013) 2. Aarikka-Stenroos et al. (2017) 3. Abbas et al. (2012) 4. Amabile (1997) 5. Bergendahl et al. (2015) 6. Bessonova and Gonchar (2017) 7. Bowles and Hattie (2013) 8. Chen et al. (2016) 9. Curran et al. (2014) 10. Demircioglu et al. (2017) 11. du Toit et al. (2011) 12. Ederer et al. (2013) 13. Galia (2008) 14. Gopal and College (2011) 15. Hartmann (2006) 16. Jain and Ali (2012) 17. Maria Stock et al. (2017) 18. Marvel et al. (2007) 19. Minarcine et al. (2016) 20. Montani et al. (2014) 21. Mudambi et al. (2007) 22. Muninger et al. (2019) 23. Öberg et al. (2014) 24. Ozorhon et al. (2017) 25. Piperopoulos, Wu, & Wang (2018) 26. Romero and Martínez-Román (2012) 27. Sergeeva and Zanello (2018) 28. Song et al. (2011) 29. Wang and Huang (2015) 30. Wang, Liu, Zhu, and Bastian (2018) 31. Wendelken et al. (2014) 32. Wu et al. (2013) 33. Yidong and Xinxin (2013)	1. Antikainen et al. (2010) 2. Cordero et al. (2005) 3. Hosseini and Narayanan (2014) 4. Jean et al. (2018) 5. Kirsten and Du Preez (2010) 6. Naidoo and Sutherland (2016) 7. Poskela and Martinsuo (2009) 8. Radicic et al. (2016) 9. Stewart Jr. et al. (1999)	1. Antikainen and Vaataja (2010) 2. Curado et al. (2018) 3. Fischer et al. (2019) 4. Jiang and Thagard (2014) 5. Manimala et al. (2006) 6. Shane et al. (2003) 7. Sorice and Donlan (2015)	1. Dee et al. (2002) 2. Edwards et al. 2014 3. Gorozidis and Papaioannou (2014) 4. Gorozidis and Papaioannou (2016) 5. Kandiko (2013) 6. Koch et al. (2015) 7. Lam et al. (2010) 8. Messmann and Mulder (2014)	1. Bolderdijk et al. (2018) 2. Costa et al. (2015) 3. Dietrich (2016) 4. Füller (2012) 5. Hopkins (2016) 6. Ng et al. (2013) 7. Kay (2011) 8. Smith and Sandberg (2018) 9. Zheng et al. (2011)	1. Almond & Power (2018) 2. Bastian et al. (2018) 3. Horkoff, Maiden, and Asboth (2019) 4. Joy (2004) 5. Kung & Chao (2019) 6. Kuznetsov and Kuznetsova (2011) 7. Mehta et al. (2008) 8. Nold (2017) 9. Olivares et al. (2013) 10. Pihie (2007) 11. Reznickova and Zepeda (2016) 12. Skinner and Drake (2003) 13. Skinner (1996) 14. Spanjol and Tam (2010) 15. Wang et al. (2011) 16. Xie and Reider (2014)

When comparing the results of analyzing articles by discipline, the disciplinary breakdown of articles mirrored the findings of Soleas (2018b) including the disproportionate representation of innovation conceptualized from business disciplines (56.09%) such as economics, management, and entrepreneurship compared to education (10.98%), higher education (10.98%), and psychology (8.54%), as well as small minorities from the public sector (7.32%), engineering (4.87%), and environmental conservation (1.22%; see Table 3 for a complete study listing by discipline).

**Table 3:** Disciplinary context of the articles

Business	Engineering	Public sector	Education	Higher education	Psychology	Environmental conservation
1. Aarikka-Stenroos et al. (2017) 2. Abbas et al. (2012) 3. Amabile (1997) 4. Antikainen et al. (2010) 5. Bergendahl et al. (2015) 6. Bessonova and Gonchar (2017) 7. Bolderdijk et al. (2018) 8. Chen et al. (2016) 9. Curado et al. (2018) 10. Curran et al. (2014) 11. du Toit et al. (2011) 12. Ederer et al. (2013) 13. Fischer et al. (2019) 14. Füller (2012) 15. Galia (2008) 16. Gopal and College (2011) 17. Hopkins (2016) 18. Jain and Ali (2012) 19. Jean et al. (2018) 20. Kay (2011) 21. Kirsten and Du Preez (2010) 22. Kung, & Chao (2019) 23. Maria Stock et al. (2017) 24. Marvel et al. (2007) 25. Minarcine et al. (2016) 26. Montani et al. (2014) 27. Mudambi et al. (2007) 28. Muninger, Hammedi, and Mahr (2019) 29. Naidoo and Sutherland (2016) 30. Ng & Feldman (2013) 31. Öberg & Shih (2014) 32. Piperopoulos, Wu, & Wang (2018) 33. Poskela and Martinsuo (2009) 34. Radicic et al. (2016) 35. Romero and Martínez-Román (2012) 36. Sergeeva and Zanello (2018) 37. Shane et al. (2003) 38. Smith and Sandberg (2018) 39. Song et al. (2011) 40. Spanjol and Tam (2010) 41. Stewart Jr. et al. (1999) 42. Wang and Huang (2015) 43. Wang et al., (2018) 44. Wendelken et al. (2014) 45. Wu et al. (2013) 46. Yidong and Xinxin (2013)	1. Cordero et al. (2005) 2. Hartmann (2006) 3. Hosseini and Narayanan (2014) 4. Ozorhon et al. (2017)	1. Aalbers et al. (2013) 2. Antikainen and Vaataja (2010) 3. Demircioglu & Audretsch. (2017) 4. Dietrich (2016) 5. Manimala et al. (2006) 6. Zheng et al. (2011)	1. Dee et al. (2002) 2. Gorozidis and Papaioannou (2014) 3. Gorozidis and Papaioannou (2016) 4. Koch et al. (2015) 5. Lam et al. (2010) 6. Messmann and Mulder (2014) 7. Pihie (2007) 8. Wang et al. (2011) 9. Xie and Reider (2014)	1. Almond & Power (2018) 2. Edwards et al. (2014) 3. Horkoff, Maiden, and Asboth (2019) 4. Kandiko (2013) 5. Kuznetsov and Kuznetsova (2011) 6. Mehta et al. (2008) 7. Nold (2017) 8. Olivares et al. (2013) 9. Reznickova and Zepeda (2016)	1. Bastian et al. (2018) 2. Bowles and Hattie (2013) 3. Costa et al. (2015) 4. Jiang and Thagard (2014) 5. Joy (2004) 6. Skinner and Drake (2003) 7. Skinner (1996)	1. Sorice and Donlan (2015)

The vast majority of studies in the sample were quantitative (71.95%), with qualitative as a sizable minority (19.51%), and mixed method studies as the rarest (8.54%; see Table 4 for a complete study listing by methodology type).

**Table 4:** Methodology type of included studies

Quantitative	Qualitative	Mixed
1. Aalbers et al. (2013) 2. Abbas et al. (2012) 3. Almond & Power (2018) 4. Amabile (1997) 5. Bastian et al. (2018) 6. Bergendahl et al. (2015) 7. Bessonova and Gonchar (2017) 8. Bolderdijk et al. (2018) 9. Bowles and Hattie (2013) 10. Chen et al. (2016) 11. Cordero et al. (2005) 12. Costa et al. (2015) 13. Curran & Walsworth (2014) 14. Dee et al. (2002) 15. Demircioglu & Audretsch (2017) 16. Dietrich (2016) 17. du Toit et al. (2011) 18. Ederer & Manso (2013) 19. Edwards et al. 2014 20. Fischer et al. (2019) 21. Füller (2012) 22. Galia (2008) 23. Gorozidis and Papaioannou (2014) 24. Gorozidis and Papaioannou (2016) 25. Hopkins (2016) 26. Hosseini and Narayanan (2014) 27. Jain and Ali (2012) 28. Joy (2004) 29. Kirsten and Du Preez (2010) 30. Koch et al. (2015) 31. Kung, & Chao (2019) 32. Kuznetsov and Kuznetsova (2011) 33. Lam et al. (2010) 34. Maria Stock et al. (2017) 35. Mehta et al. (2008) 36. Messmann and Mulder (2014) 37. Montani et al. (2014) 38. Mudambi et al. (2007) 39. Nold (2017) 40. Olivares et al. (2013) 41. Ozorhon et al. (2017) 42. Pihie (2007) 43. Piperopoulos, Wu, & Wang (2018) 44. Poskela and Martinsuo (2009) 45. Radicic et al. (2016) 46. Romero and Martínez-Román (2012) 47. Shane et al. (2003) 48. Skinner and Drake (2003) 49. Skinner (1996) 50. Song et al. (2011) 51. Spanjol and Tam (2010) 52. Stewart Jr. et al. (1999) 53. Wang and Huang (2015) 54. Wang et al. (2011) 55. Wang et al. (2018) 56. Wu et al. (2013) 57. Xie and Reider (2014) 58. Yidong and Xinxin (2013) 59. Zheng et al. (2011)	1. Aarikka-Stenroos et al. (2017) 2. Antikainen et al. (2010) 3. Gopal and College (2011) 4. Hartmann (2006) 5. Jiang and Thagard (2014) 6. Kandiko (2013) 7. Kay (2011) 8. Manimala et al. (2006) 9. Marvel et al. (2007) 10. Minarcine & Shaw (2016) 11. Naidoo and Sutherland (2016) 12. Reznickova and Zepeda (2016) 13. Sergeeva and Zanello (2018) 14. Smith and Sandberg (2018) 15. Sorice and Donlan (2015) 16. Wendelken et al. (2014)	1. Antikainen and Vaataja (2010) 2. Curado et al. (2018) 3. Horkoff, Maiden, and Asboth (2019) 4. Jean et al. (2018) 5. Muninger, Hammedi, and Mahr (2019) 6. Ng & Feldman (2013) 7. Öberg & Shih (2014)

In terms of research design, surveys were by far the most common design (62.19%), followed by case studies (13.41%), experimental and quasi-experimental designs (9.75%), interviews (9.75%), and then meta-analyses (4.88%; see Table 5 for a complete listing by design).

**Table 5:** Research design of included studies

Survey	Case study	Meta-analysis	Interview	Experimental/quasi-experimental
1. Aalbers et al. (2013) 2. Abbas et al. (2012) 3. Amabile (1997) 4. Bergendahl et al. (2015) 5. Bowles and Hattie (2013) 6. Chen et al. (2016) 7. Cordero et al. (2005) 8. Curado et al. (2018) 9. Curran & Walsworth (2014) 10. Dee et al. (2002) 11. Demircioglu & Audretsch (2017) 12. Dietrich (2016) 13. du Toit et al. (2011) 14. Fischer et al. (2019) 15. Füller (2012) 16. Galia (2008) 17. Gopal and College (2011) 18. Gorozidis and Papaioannou (2014) 19. Gorozidis and Papaioannou (2016) 20. Hopkins (2016) 21. Jain and Ali (2012) 22. Joy (2004) 23. Koch et al. (2015) 24. Kuznetsov and Kuznetsova (2011) 25. Lam et al. (2010) 26. Maria Stock et al. (2017) 27. Mehta et al. (2008) 28. Messmann and Mulder (2014) 29. Mudambi et al. (2007) 30. Nold (2017) 31. Olivares et al. (2013) 32. Ozorhon et al. (2017) 33. Pihie (2007) 34. Piperopoulos, Wu, & Wang (2018) 35. Poskela and Martinsuo (2009) 36. Radicic et al. (2016) 37. Romero and Martínez-Román (2012) 38. Skinner and Drake (2003) 39. Skinner (1996) 40. Song et al. (2011) 41. Stewart Jr. et al. (1999) 42. Wang et al. (2018) 43. Wu et al. (2013) 44. Xie and Reider (2014) 45. Yidong and Xinxin (2013) 46. Zheng et al. (2011) 47. Montani et al. (2014) 48. Bessonova and Gonchar (2017) 49. Hosseini and Narayanan (2014) 50. Antikainen and Vaataja (2010) 51. Wang and Huang (2015)	1. Aarikka-Stenroos et al. (2017) 2. Antikainen et al. (2010) 3. Hartmann (2006) 4. Horkoff, Maiden, and Asboth (2019) 5. Jiang and Thagard (2014) 6. Manimala et al. (2006) 7. Muninger et al. (2019) 8. Öberg & Shih (2014) 9. Smith and Sandberg (2018) 10. Sorice and Donlan (2015) 11. Wendelken et al. (2014)	1. Almond & Power (2018) 2. Costa et al. (2015) 3. Ng & Feldman (2013) 4. Shane et al. (2003)	1. Minarcine & Shaw (2016) 2. Naidoo and Sutherland (2016) 3. Jean et al. (2018) 4. Kandiko (2013) 5. Kay (2011) 6. Marvel et al. (2007) 7. Reznickova and Zepeda (2016) 8. Sergeeva and Zanello (2018)	1. Bastian et al. (2018) 2. Bolderdijk et al. (2018) 3. Edwards et al. (2014) 4. Ederer & Manso, (2013) 5. Kirsten and Du Preez (2010) 6. Kung & Chao (2019) 7. Spanjol and Tam (2010) 8. Wang et al. (2011)

## Results for expectancies in the literature: Can I do this?

Innovation stands as an interesting case for EVC as the factors that might potentially motivate innovation are numerous. This study organizes the findings into the EVC framework to cluster together adjacent strategies that would focus on building learner confidence to innovate. In terms of the expectancies (self-efficacy and self-concept; see Bandura, 1986, 2001, 2006; Wigfield, 1994), persons who grow to see themselves as potentially able to innovate because of an acquired efficacy or confidence would typically hold a higher expectation of themselves to be able to innovate and. thus, become more invested in the task of innovating. Higher investment in the task results in a higher degree of motivation sourced from the self-held conviction that the individual can innovate. This theme focused on the strategies used by leaders to help learners engage in the task so that they could build their confidence. This establishes a foothold for further growth, as a growing expectancy of success begets further growth of the skill in innovation and elsewhere. This theme consolidates the research pertaining to the building of confidence and the strategies that a leader could integrate into their decision-making to better stoke innovation. Leaders and managers in organizations assigning simpler innovation tasks early in a program and then building to harder problems according to this logic line should steadily build esteem and self-efficacy.

### Strategies for building expectancies

Researchers have considered a wide range of expectancy-building strategies to promoting innovation and have studied the effects that careful managerial planning, engagement seeking, experimentation, teaching the process of innovating, management style, collaboration, available support, and training would have on the confidence and self-efficacy of innovators. The research on strategies for motivating innovation tended to be specific to disciplines and typically use survey methodologies of workers in businesses rather than specifically probing the motivations of innovators. Leader strategies include careful managerial planning, encouraging experimentation, managerial style, fostering collaboration, and providing supports.

#### Careful planning and engagement seeking

The literature on promoting innovation tended to come from business settings and proposed that a key consideration for actively stoking confidence to innovate was careful managerial planning, with specific consideration of consistent efforts maximizing engagement opportunities. These efforts were found by some studies to be most fruitful when they led to informal or even improvised activities (Song, Im, Van Der Bij, & Song, 2011). Monge et al. (1992) found that careful managerial planning of meetings as well as cognizance of the peaks and troughs throughout yearly or periodic cycles were necessary; otherwise, employers risked shorting the confidence of participants to innovate. Similarly, Messmann and Mulder (2014) found that managers need to carefully consider that no matter how innovative a person might be over time, innovation fatigue can set in unless confidence is sufficiently stoked.

Research has identified the importance of careful managerial planning as key to the maintenance and encouragement of worker engagement and thereby confidence, such as information days, group goal setting, and stakeholder consultations (Hartmann, 2006; Monge et al., 1992; Pihlajamaa, 2017; Smith & Sandberg, 2018). Studies have shown that carefully curated interactions with others and having project teams that were crafted to include a variety of expertise tended to increase confidence in innovating (Marvel et al., 2007; Song et al., 2011). Other studies found that the organizational choices of leaders were also impactful in boosting innovation confidence (Almond & Power, 2018; Dee et al., 2002; Smith & Sandberg, 2018). Montani et al. (2014) pointed to the need for managers to provide practical tools and structures for these engagement efforts such that they became habitual and a part of organizational muscle memory. In short, managers and those in supervisory roles need to take explicit and thoughtful action to build and sustain the confidence to innovate of those in their employ.

#### Leader encouraging experimentation and innovators learning through experience

Research that examined strategies that built innovation confidence through the encouragement of experimentation tended to come from business settings and primarily consisted of survey studies. Demircioglu and Audretsch (2017) found that providing opportunities for experimentation coupled with feedback loops in work situations tended to build the expectancies of workers to innovate. Other studies found that experimentation was facilitated through the work environment and by the flexible allocation of work tasks that tolerated early failures (Almond & Power, 2018; Ederer & Manso, 2013; Messmann & Mulder, 2014; Muninger et al., 2019. These same studies found that this early investment of time and resources for pilot testing or at least tolerance of early mistakes overwhelmingly tended to yield long-term success in excess of less tolerant attitudes towards experimentation.

In the same vein, problem finding, problem clarification, and problem-setting were found to be effective catalysts for experimentation (Füller et al., 2012; Montani et al., 2014; Susha et al., 2015). These initiatives were commonly found to be most effective when coupled with systematic evaluation strategies (Messmann & Mulder, 2014; Pihie, 2007). Demircioglu and Audretsch (2017) found that experimentation spurred innovative activity because it suited employee preference to feel in control over their actions. This recognition of the desire of innovators to be self-determining is congruent with the findings of decades of self-determination theory research (e.g., Deci & Ryan, 1987; Ryan & Deci, 2017). In summary, the contemporary thinking on encouraging experimentation is that it can be coupled with preliminary thinking exercises, consistent feedback, and then post-experimental systematic evaluations to be maximally effective in building innovators’ confidence, and thereby supporting innovation.

#### Leadership style: the effects of having innovation champions

Research within the business field has identified leadership style and the efficacy of champions as key-innovation confidence-building tools. Leaders who boldly pursued innovation (Hsu, 2009; Wang et al., 2018), ensured that a variety of employees and resources were brought to bear on a given challenge (Aarikka-Stenroos et al., 2017), held a tolerance for ambiguity (Koch et al., 2015; Shane et al., 2003), and, otherwise, made space temporally, attitudinally, and organizationally for innovation was effective in building the confidence of their work forces (Song et al., 2011). This mirrors findings elsewhere that transformative leadership (known colloquially as visionary leadership; Kandiko, 2013) was effective in making innovation more likely (Abbas et al., 2012; Bolderdijk et al., 2018; Sergeeva & Zanello, 2018; Y. Wang et al., 2018; Yidong & Xinxin, 2013). These same leaders were found to diminish the deleterious effects of external rewards by setting the focus on the interest of the task and teamwork (Bolderdijk et al., 2018; Chen, Li, & Leung, 2016; Yidong & Xinxin, 2013). Amabile (1997) in particular described this as leaders who nurture the spark of innovation and who would build confidence at the same time as they diminished perceived costs. These costs sometimes involved innovation taking the form of an emotional roller coaster: effective champions temper or incite enthusiasm when the situation calls for it (Pihlajamaa, 2017).

Similarly, leaders or managers who supported innovation tended to take a promotion focus (playing to win; Poskela & Martinsuo, 2009), rather than a loss prevention focus (playing not to lose; Manimala, Jose, & Thomas, 2006; Poskela & Martinsuo, 2009), thereby taking calculated risks and enabling their followers to do the same (Bowles & Hattie, 2013; Koch et al., 2015; Maria Stock, Zacharias, & Schnellbaecher, 2017; Sergeeva & Zanello, 2018; Spanjol & Tam, 2010). While a majority of studies found that the effect of champions was significant, Chen, Li, and Leung (2016) pointed out that two recent meta-analyses revealed that champions only had a small effect on the quantity of innovation by individuals. Naidoo and Sutherland (2016) gave nuance to this finding by determining in their sampling that the reported quality of innovations was increased rather than quantity when groups were led by champions. In summary, leaders who promoted confidence to innovate tended to provide the resources and spaces for innovation to occur and tended to lead efforts personally and model innovative behavior rather than delegating.

#### Collaboration

By far, the most common theme among analyzed research reports and papers and across disciplines was the widespread finding that collaboration served as a strategy for promoting innovation through building confidence (e.g., Bastian et al., 2018; Bergendahl et al., 2015; Curado et al., 2018; Curran & Walsworth, 2014; Fernandez & Pitts, 2011; Pihlajamaa, 2017). Effective collaboration was found to increase expectancies for innovation though necessitating the communication and subsequent discussion of ideas (Bastian et al., 2018; Curado et al., 2018; Fernandez & Pitts, 2011; Hartmann, 2006; Jiang & Thagard, 2014; Monge et al., 1992; Pihlajamaa, 2017). Collaboration was also found to involve diverse stakeholders (Aarikka-Stenroos et al., 2017; Costa et al., 2015; Kuznetsov & Kuznetsova, 2011; Sergeeva & Zanello, 2018; Wu et al., 2008), facilitate empowerment (Bolderdijk et al., 2018; Fernandez & Pitts, 2011), promote knowledge exchange (Aalbers et al., 2013; Antikainen & Vaataja, 2010; Bastian et al., 2018; Curado et al., 2018; Muninger et al., 2019; Naidoo & Sutherland, 2016), facilitate co-designing (Hartmann, 2006; Sorice & Donlan, 2015), inspire friendly competition between teams (Lam et al., 2010; Naidoo & Sutherland, 2016; Öberg & Shih, 2014), as well as help team and coalition building which ensure that a person does not have to go it alone (Costa et al., 2015; Dee et al., 2002; Kirsten & Du Preez, 2010; Mc Fadden & Gorman, 2016; Öberg & Shih, 2014). These outcomes of collaboration, such as networking (Mc Fadden & Gorman, 2016), all induced increases in confidence to innovate, especially in female entrepreneurial networks (Apergis & Pekka-Economou, 2010). Other studies chose to rely on outcome measures, such as Galia (2008) who found that the more innovative firms in their sample tended to make extensive use of teams, and Antikainen et al. (2010), who pointed to community satisfaction based on the inclusion on projects through collaboration.

While research has identified the significant benefits and articulated some risks of misusing collaboration, studies have shown that team chemistry is a key consideration in reaping the maximal benefit of collaboration to motivate innovation. Manimala et al. (2006) identified that loose or informal team formation decreased the confidence of the individuals in a group as well as the efficacy of the group. As well, striking a balance of collaboration and competition in a given work environment was seen as a way to make the most of team work while also encouraging accountability (Naidoo & Sutherland, 2016). Mehta et al. (2008) found that while it is often best to have a mixture of motivations and attitudes on teams when working under strict timelines or with budgetary concerns, having homogenous groups is typically the better decision. Indeed, actor diversity helps to ensure that many ideas are represented when working in teams (Aarikka-Stenroos et al., 2017; Bastian et al., 2018; Bolderdijk et al., 2018; Dietrich et al., 2016; Kung & Chao, 2019; Pihlajamaa, 2017). Pihlajamaa (2017) extends actor diversity to recommend that teams be composed of innovators and non-innovators in a hammer and anvil model, where innovators strike ideas and non-innovators provide support and stability. In summary, contemporary research on collaboration as a tool to support innovation has identified that it has a wide range of significantly impactful benefits but needs to be applied with caution, as too much dependence on collaboration can result in a decrease of accountability. In addition, optimal team composition is extremely sensitive to the personalities of the members and the goals of the innovation.

#### Support

Support was a consistent consideration of research examining strategies that build expectancies and confidence to innovate (e.g., Kuznetsov & Kuznetsova, 2011). Common supports identified included creating a conducive innovation climate or culture (Dee et al., 2002; Hopkins, 2016; Kung & Chao, 2019; Montani et al., 2014; Susha et al., 2015), having reliable infrastructure (Susha et al., 2015), availability of mentoring (Apergis & Pekka-Economou, 2010), and inviting folks to engage with groups pursuing innovation (Apergis & Pekka-Economou, 2010; Curado et al., 2018; Messmann & Mulder, 2014; Radicic, Pugh, Hollanders, Wintjes, & Fairburn, 2016; Susha et al., 2015). Explicitly modelling flexibility and exploration (Amabile, 1997; Hartmann, 2006; Montani et al., 2014; Sorice & Donlan, 2015), breaking a task down into smaller, manageable pieces (Dietrich et al., 2016; Pihie, 2007), and providing ample time and financial resources (Aarikka-Stenroos et al., 2017; Hartmann, 2006; Hosseini & Narayanan, 2014; Ozorhon & Oral, 2017) were also found to be crucial supports for confidence to innovate.

Studies also examined the importance of feedback as a support that increased individual and group confidence when innovating (Demircioglu & Audretsch, 2017; Ford, 1999; Hartmann, 2006). Other supports included innovation policies (Ozorhon & Oral, 2017), active inclusion of a problem-solving curriculum (Pihie, 2007), and actively enforcing a high quality of life balance (Minarcine & Shaw, 2016; Sorice & Donlan, 2015).

In summary, supports were widely positioned as methods or tools to build confidence and heuristics to make innovation as a process easier; these supports tended to divide innovation into manageable segments, scaffold the process of innovating, and, otherwise, decrease the costs of innovation. These strategies were typically identified in the discussions of studies, rather than as the subjects of studies, and often lacked a concrete explanation as to what these supports would look like. For example, while an innovation supportive culture, climate, or policy was often identified as a necessary factor in building confidence to innovate, explanation and articulation of what constituted such a culture, climate, or policy remained sparsely defined.

## Results for perceived values: What makes innovation worth doing?

Individuals tend to act in accordance with the perceived values that they see in the tasks at hand (Feather, 1992). The three value types such as intrinsic, attainment, and utility subjective task value lend themselves well to an analysis of a phenomenon like innovation, as it is perceived differently by different people with different values (Green, 2013). For instance, individuals might have a very different attainment valence than their peers regarding innovation. Some might see innovation as having a very high attainment value as it is of central importance to their sense of self or in constructing their identity. Similarly, innovation as an act may hold intrinsic value to some people as they enjoy the act of applying their ideas. People tend to be motivated to complete activities that they enjoy; hence, if they enjoy the task of innovating and its subtasks they are likely to continue to do so (intrinsic value). But what if an individual does not enjoy innovation yet still is motivated? This motivation might be a utility value. Even if people think they can innovate, they might not have the motivation to innovate unless they see what is in it for them. Innovation is often richly rewarded in society, even if many think innovation is a buzzword (Green, 2013; Lehmann-Ortega & Schoettl, 2005). This reward can be in the form of monetary pay, public recognition, and fame, although it varies widely by context. The lure of these rewards can beget a value for which individuals can strive, giving the act of innovation an inherent value for the individual who seeks these rewards. Of all these forms of value motivation, intrinsic is hypothesized to be the most powerful and most desired across a majority of contexts (Deci et al., 1999); however, the relative potency varies from individual to individual.

### Intrinsic task value

Intrinsic task value is the subjective value of a task for enjoyment, interest, or fun (Barron & Hulleman, 2015; Eccles & Wigfield, 2002). Innovative activity has been known to have intrinsic value to individuals if they find the act enjoyable or interesting (Amabile & Kramer, 2011; Fischer et al., 2019). Research that addressed strategies that increased intrinsic task value were rare in the literature; instead, research tended to focus on tasks and their presentation to individuals.

#### Strategies for building intrinsic value

The lone strategies found in the literature were to foster the interests and encourage the drive of individuals who undertook innovative endeavours.

##### ***Interest, enjoyment, and novelty seeking as means to create drive***

The few strategies that studies examined pertaining to stoking innovation through intrinsic task value focused solely on feeding individuals’ interest and drive. Strategies that motivated innovation tended to be enjoyable and, hence, very much specific to the interest of the participants (e.g., Antikainen & Vaataja, 2010; Bolderdijk et al., 2018; Galia, 2008; Wendelken et al., 2014). Powerful manifestations of this motivation included informal and recreational settings (Antikainen & Vaataja, 2010), flexibility of choice (Cordero et al., 2005; Fischer et al., 2019), efforts to make otherwise dull tasks enjoyable (Wendelken et al., 2014), and participating in events and programs with the focus of being enjoyable (Bolderdijk et al., 2018; Fischer et al., 2019; Zheng et al., 2011). The findings of the studies point to efforts to make meetings, programs, and work settings to be enjoyable through curated informal settings. Additionally, understanding that flexibility of choice and leader-led efforts to make tasks enjoyable tended to increase the intrinsic task value of a given task.

Personal interest and curiosity were found to be powerful motivators for innovation (Amabile, 1997; Bolderdijk et al., 2018; Fischer et al., 2019; Füller et al., 2012; Minarcine & Shaw, 2016; Öberg & Shih, 2014; Susha et al., 2015). When tasked with deriving new ideas, few other motivations matched the potency of being truly interested in the task (Amabile, 1997; Füller et al., 2012). In fact, interest was found to be one of the few intrinsic motivations that extrinsic motivation could not quell (Amabile, 1997; Minarcine & Shaw, 2016; Öberg & Shih, 2014). In summary, wherever possible, efforts should be made to strategically allocate personnel to tasks that they would find interesting.

Strategies that supported innovation were found to feed the novelty-seeking behavior of many innovators. Studies suggested task variety (Duverger, 2012; Montani et al., 2014), thrilling goals (Joy, 2004), imaginative involvement (Joy, 2004), and new experiences (Edwards et al., 2014; Joy, 2004; Wang et al., 2011) as motivating innovation. Leaders making the efforts to design their strategies to provide these opportunities tended to fulfil intrinsic task value through fulfilling the “need for new” that many innovators feel.

### Attainment value

Attainment task value is the value perceived as a result of completing a task; this aligns with the internal extrinsic motivation of the Self-determination theory (Ryan & Deci, 2017).

#### Strategies for building attainment value

Attainment value was a frequent finding in the literature on motivation to innovate. Common trends for promoting attainment value included strategies like fostering autonomy, investment, recognition, optimized challenge, and perceived importance of innovative endeavors.

##### ***Autonomy***

Strategies for building autonomy focused on judicious delegation of decision-making processes and allowing workers to make planning decisions (Costa et al., 2015; Hartmann, 2006). Another tactic was encouraging followers to develop with new ideas and put them into practice as a means of fuelling initiative (Yidong & Xinxin, 2013). Shane et al. (2003) argued that initiative building facilitated the added benefit of encouraging workers to think of themselves as being capable, and this made innovation seem more valuable. While articles highlighted that providing autonomy was a way to increase the attainment value of innovation, the exact mechanism does not seem to be fully understood beyond the indication that giving people choice makes them more likely to commit to their new ideas.

##### ***Investment***

Studies pointed to strategies that built investment to be highly effective in promoting innovation. These strategies included decision-making being proportional to the amount of investment in corporations where the board held a controlling interest in the company (Bessonova & Gonchar, 2017). When boards made up of employees held a controlling interest, innovation was more common than when they did not. Similar non-corporate situations where the decision makers were personally and sometimes monetarily invested in the outcome of an endeavor tended to also be more likely to support innovation (Bessonova & Gonchar, 2017; Hartmann, 2006; Wendelken et al., 2014). In situations where individuals were personally invested in their endeavors, innovation tended to increase.

##### ***Recognition***

Recognition, past, present, and future was found to be an important consideration when making efforts to motivate innovating. Research has shown that motivation to gain reputation was a central consideration of many aspiring innovators in the public domain (Fischer et al., 2019; Zheng et al., 2011), and in the private business (Antikainen & Vaataja, 2010; du Toit, van Staden, & Steyn, 2011) and for teachers implementing problem-solving initiatives in their classrooms (Lam et al., 2010). This consideration was articulated as a combination of recognition of status, peer-respect dynamics, and enhancement of professional status (Fernandez & Pitts, 2011; Wendelken et al., 2014). Recognition of innovative efforts in the literature was split between being reported as a decisive factor in some articles, while not being a great consideration in others indicating that further study is needed to determine the dynamics mediating other factors.

##### ***Self-improvement, achievement, and optimized challenge***

Research has also examined strategies for motivating innovation that involved feeding the desire of aspiring innovators to improve themselves. This strategy has been described as feeding a self-improvement drive (Aarikka-Stenroos et al., 2017; Antikainen & Vaataja, 2010; Piperopoulos et al., 2018) or as a hunger to take on new challenges (Edwards et al., 2014; Jain & Ali, 2012; Lopez & Snyder, 2003; Marvel et al., 2007). “Hunger” literature tended to portray innovators as constantly looking for what others say cannot or should not be done and are engaged when experiencing the right balance of challenge and support. This Vygotskian idea (Glassman, 2001; Vygotsky & Kozulin, 2011) is also inferred in the self-improvement literature through self-scaffolding and a leader’s frequent and tactical provision of skill development opportunities for employees.

##### ***Importance, relevance, and clear need***

The dominant attainment task value as gleaned from the literature is the importance, relevance, and clear need for innovative behavior and goals (Aarikka-Stenroos et al., 2017; Hosseini & Narayanan, 2014). To this end, studies suggested that clear links to desired goals like careers or mastery (Aarikka-Stenroos et al., 2017; Edwards et al., 2014; Sorice & Donlan, 2015; Xie & Reider, 2014) and easy to see logic to how the innovation would be important to society, with a particular emergent theme of social justice and conservation causes (Antikainen & Vaataja, 2010; Pihie, 2007; Reznickova & Zepeda, 2016; Sorice & Donlan, 2015). Leaders seeking to stoke innovation could infer from the literature that making the time to articulate the goals of an innovation and the way that it could benefit workers and society will increase engagement and increase the attainment value of the activity thus propelling the innovating of individuals and teams.

### Utility value

#### Strategies for building utility value

The lone strategy explored in the literature to build utility value was through the medium of rewards. Rewards were, however, an extremely common theme in the literature being explicitly and implicitly examined in a majority of studies.

##### ***Rewards***

Studies highlighted that offering monetary rewards for innovation encouraged participation in innovating collaborations (Aarikka-Stenroos et al., 2017; Antikainen & Vaataja, 2010; Fischer et al., 2019; Galia, 2008; Hartmann, 2006; Marvel et al., 2007; Smith & Sandberg, 2018; Susha et al., 2015; Zheng et al., 2011). Kandiko (2013) found that in higher education contexts, external rewards like grants were seen as necessary evils that created the space to innovate. Thus, the reward was a means to an end and not the desired outcome itself. The results of other studies supported this view of rewards as tools for creating space and opportunities for innovating (Wendelken et al., 2014; Xie & Reider, 2014). However, other studies pointed to the lack of empirical evidence on types of rewards outside of business case studies (Kay, 2011). The few studies on the empirical evidence of rewards argued that the most effective rewards tended to not be immediate in time, or directly in the form of monetary rewards (Baranchuk et al., 2014; Kay, 2011). Rather, rewards with a longer vesting period or with a longer duration before payoff tended to be more effective (Baranchuk et al., 2014; Lerner & Wulf, 2018). As a final level of complexity, Kay (2011) and Amabile (1997) found that the timing of rewards made a dramatic difference in the success of the innovation with early funding and rewards being much more effective in propelling innovations to success.

##### ***Indirect rewards work better than direct rewards***

The findings of some studies offer nuances to this understanding, reflecting that while monetary rewards and other extrinsic motivators generally worked at some level, they were not as necessary as other motivations like career aspirations, fulfillment, interest, or non-monetary rewards. These in tandem with changes to organization or administration were considered to be more influential (Costa et al., 2015; Kandiko, 2013; Sorice & Donlan, 2015; Thapa et al., 2015; Wendelken et al., 2014). Curran and Walsworth (2014) found that pay-for-performance or high salaries were ineffectual in stoking the motivation to innovate, whereas indirect pay such as benefits or group pay increased motivation to innovate, findings corroborated elsewhere in the literature (Ederer & Manso, 2013; Lerner & Wulf, 2018; Ng & Feldman, 2013). The lone study to make concrete recommendations for indirect rewards was Hartmann (2006) who found that family health benefits, excursions, additional resources for their goal, and allowances for professional development were effective indirect rewards. Similarly, to the short-term rewards, performance pay was found to induce the feeling of being controlled, instigating decreased motivation to innovate; whereas, long-term goals created autonomous motivation (Ederer & Manso, 2013).

##### ***Variety of rewards***

Further still, some studies found that outcome rewards in any form were detrimental to the motivation to innovate (Antikainen et al., 2010; Poskela & Martinsuo, 2009). These other studies’ results tended to find that including a variety of rewards was the best strategy if rewards were necessary (Bessonova & Gonchar, 2017; Cordero et al., 2005; Maria Stock et al., 2017). Curran and Walsworth (2014) found that it was sometimes possible to increase innovation with compensation, but only with the correct compensation, which connects to the arguments of Hopkins (2016), who calls for material, communal, or related incentives to foster innovative behavior. Hartmann (2006) analogously found that the major predictor of the efficacy of rewards was having a variety of rewards, findings echoed in those of other studies (Amabile, 1997; Cordero et al., 2005; Hopkins, 2016). In a study of innovation for participants and non-participants, the non-participants placed the most emphasis on monetary rewards, while the innovator participants placed more emphasis on other more internal motivators (Wendelken et al., 2014). In summary, there is substantial evidence in the literature that monetary rewards may not attract the desired motivations, and that the best course of action would be to offer a variety of rewards.

## Results for cost in the literature: **What is between me and what I want?**

Whereas, expectancies and values are promotive constructs, costs are the hindering factors that make a task, in this case, innovation, less likely. The strategies discussed here are for mitigating the costs of innovating, such that there is more room for leaders to use expectancy and value-building strategies to motivate innovation. Diminishing the potential intrinsic, attainment, and utility value of innovating is the inherent costs of being an innovator. Innovation, like other complex tasks, while potentially rewarding, also has contextual material and psychological costs, such as additional effort, investment of time, pressure, the implications of failure, and loss of both relative stability from the status quo and availability of other options (Flake et al., 2015). For instance, the process of innovating may very well require the investment of additional time and resources to design and operationalize. However, doing things as they have been done in the past does not. The cost of the additional resources may serve to lessen the motivation or diminish the value of innovating. Innovation may itself place the individual or collaboration under pressure that may be undesirable for some individuals (Flake et al., 2015; Vansteenkiste et al., 2005; Wang et al., 2018). Innovation has been portrayed as a risky pursuit because of the possibility of failure, the stigma of being different, and a threat to the status quo (Green, 2013; Lehmann-Ortega & Schoettl, 2005). Even if someone innovates, the idea might not hold the same value to other people. Innovation does have a cost. To some, it constitutes the loss of non-innovative alternatives. To promote innovation development, the expectancies and values must exceed the costs, and this balancing act may be facilitated by the use of strategies or the design of environments.

### **Strategies for managing costs**

In the literature, successful strategies for motivating innovation considered the costs of innovation including risk aversion and unbalanced focus on financial rewards. Costs are, by far, the least defined and studied strand from an EVC perspective on motivating innovation. Strategies informed and actively designed to mitigate the costs of innovation as identified in the findings of studies tended to be the most successful.

#### Risk aversion

Studies promoting innovation demonstrated that a crucial conundrum is the mitigation of the perception of risk; innovation is seen as a risky thing by many aspirants (Kinney et al., 2015; Phillips, 2004; Pihie, 2007). Study results pointed to risk as being needed to be managed by leaders for innovative behavior to occur (Poskela & Martinsuo, 2009; Skinner & Drake, 2003; Stewart Jr. et al., 1999; Wang & Huang, 2015). Another path to the same idea, the promotion of risk acceptance was seen as a way to combat the corrosive effects of risk on the motivation to innovate (Manimala et al., 2006; Marvel et al., 2007; Messmann & Mulder, 2014; Shane et al., 2003). Marvel et al. (2007) provided nuance to this understanding by showing that lack of risk aversiveness is shown to decrease motivation; however, risk acceptance was not shown to be particularly motivating. Risk acceptance is only visible in its absence; hence, it is a cost mitigation factor rather than an expectancy. Research into the effects of risk on innovation was limited to directionality and the consensus is that the magnitude of risk deters innovative behavior. However, this synthesis offers little insight into the strategies that leaders could use to mitigate the impact of risk.

#### Obsession with financial rewards

While financial incentives were hypothesized to have some positive benefits such as increasing interest, an organization or group with too much of a focus on monetary rewards actually increases the cost of innovating as a result of the cutthroat behavior of some workers (Sorice & Donlan, 2015). To combat this, extrinsic rewards once utilized need to be used with increasing frequency and magnitude (Bessonova & Gonchar, 2017; Maria Stock et al., 2017; Sorice & Donlan, 2015) in a manner that seems reminiscent of a pattern of addiction. To have the same effect, increased amounts of rewards needed to be utilized to sustain the pattern of behavior.

## Discussion

It is clear that there is an unbalanced primacy in the innovation literature in favor of business and corporate settings with very little representation from the arts or social justice sectors. There is also a common trend of using surveys of individuals within a single discipline, while interviews are rare. Although there is an emergent field of study on innovation education, this field has yet to consider the unique motivation of aspiring innovators. At the moment, innovation promotion is still very much disciplinary and siloed as shown by the complete absence of interdisciplinary research on environments or strategies. The gaps in understanding the effects of various environments and strategies are detrimental to efforts to support innovation. The paucity of studying costs of innovation in the literature is symptomatic of the primarily positive psychology approach taken by studies, rather than a framework like EVC which also looks at detractive factors like costs. Efforts to support innovation in a variety of settings including education need to have a deeper understanding of the costs and prices paid by innovators so that they can be mitigated and addressed.

In terms of methodology, the field is dominated by surveys of employees in business settings, rather than of bonafide innovators. This focus on survey research precludes the possibility of having concrete details and rich articulation of narration from innovators on their thinking. Another manifestation is the lack of specific strategies across the strategies and environments as the literature tends to focus on measurable outcomes rather than the latent considerations that underpin the decisions that aspiring innovators make and the supports and barriers that they consider. The literature offers many ideas about what motivates innovation, but there has been very little open-ended investigations directly asking innovators what factors motivated them to reach for their prospective goals, nor are innovators effectively asked about their education. For this reason, additional research is required to investigate the specific motivations of innovators in a variety of disciplines.

Numerous studies provide support for the notion that more internal motivations like intrinsic (e.g., interest) and attainment (e.g., importance, fulfilment) were more influential than external motivators like rewards as targets of strategies (Aarikka-Stenroos et al., 2017; Fernandez & Pitts, 2011). In an experimental study, Ederer and Manso (2013) found that pay for performance actively inhibited innovation precursor behaviors such as exploration and resulted in significantly fewer innovative events as compared to conditions where exploration was rewarded. Namely, internal motivations were more effective as they were not seen as being as controlling as external rewards showing that individuals tend to see through direct rewards and recognize them as forms of external control (Aarikka-Stenroos et al., 2017; Fernandez & Pitts, 2011; Galia, 2008; Gorozidis & Papaioannou, 2016; Hartmann, 2006; Jermias, 2007). That being said, the more related the reward is to the activity itself in terms of subject matter, the more effective the reward is in promoting the desired behavior (Cordero et al., 2005; Fernandez & Pitts, 2011). Rewarding someone who enjoys reading by giving them money would reduce internal motivation and might actually decrease the amount they read, as compared to rewarding someone for reading by giving them another book. These findings are supported by a history of research in the motivation literature (e.g., Kruglanski et al., 1975; Kruglanski et al., 1971; Ryan & Deci, 2017)^,^ showing that the nascent research into reward effect on promoting innovation has research precedent elsewhere.

Leaders were urged to focus on topics that engaged curiosity, interest, and satisfaction (Antikainen et al., 2010; Kandiko, 2013; Shane et al., 2003; Wang & Huang, 2015; Wu et al., 2013). These strategies recommended that leaders only assign topics to innovators that were of interest to them. Such strategies would not be applicable in many situations where the topic is not flexible. Additional research is needed to determine how intrinsic task value can be applied in situations where topics are not intrinsically interesting for innovators as well as to determine the mechanisms of intrinsic value and how it works to motivate innovation.

Forward-thinking companies exemplified in the literature had moved away from direct rewards like monetary bonuses and had instead moved towards recognition and indirect rewards like vacation time, paying for the professional development of the employee’s choosing, and flexible work time (Curran & Walsworth, 2014; Gopal & College, 2011). The general trend among studies is that internal motivations and rewards that have longer vesting times or feed interest or attainment operate effectively long-term, while external motivations like direct rewards or pay for performance tend to have short-term effects that are reliant on consistent rewarding of behavior and can even actively hinder innovation. Leaders, if they choose to use rewards, should focus their strategies to give related rewards; otherwise, they risk sundering the internal motivation to innovate for already interested workers.

## Conclusion

This manuscript consolidates the available knowledge from for leader-driven strategies for motivating innovation. By doing so, it provides avenues for discussion and ideas for implementation by leaders in context who seek to promote innovation. It identifies patterns from a range of disciplines to break the siloing of knowledge from different innovation-related areas of endeavor and makes it available for innovation promotion efforts. The most effective leader approaches to making innovation more likely combine maximizing promotive factors while also addressing and mitigating hindering factors. Effective leaders in organizations actively maximize confidence of their workers and account for the values that they see in innovating. These effective leaders in conjunction with approaches that promote innovating make decisions that mitigate or reduce the perceived costs of innovating. The optimal use of rewards in the workplace and learning setting are related to the task and are often not direct performance pay. This work points to the most effective approaches to motivating innovation being those that proactively build expectancies and values, while continuously working to mitigate the costs of innovating.

## Data Availability

Data sharing is not applicable to this article as no datasets were generated or analyzed during the current study. However, lists of analyzed articles are available in the manuscript.

## Abbreviation

EVC: Expectancy–value–cost theory (Barron and Hulleman, 2015). A major theory of motivation that theorizes that the motivation for a task is the function of the expectancies (expectations of success, confidence, and self-efficacy) and the values (a direct or indirect, tangible or intangible benefit for performing the task) balanced against the perceived costs of performing the task.

## References

[CR1] Aalbers R, Dolfsma W, Koppius O (2013). Individual connectedness in innovation networks: on the role of individual motivation. Research Policy.

[CR2] Aarikka-Stenroos L, Jaakkola E, Harrison D, Mäkitalo-Keinonen T (2017). How to manage innovation processes in extensive networks: a longitudinal study. Industrial Marketing Management.

[CR3] Abbas G, Iqbal J, Waheed A, Riaz MN (2012). Relationship between transformational leadership style and innovative work behavior in educational institutions. Journal of Behavioural Sciences.

[CR4] Almond K, Power J (2018). Breaking the rules in pattern cutting: an interdisciplinary approach to promote creativity in pedagogy. Art, Design & Communication in Higher Education.

[CR5] Amabile TM (1997). Motivating creativity in organizations: on doing what you love and loving what you do. California Management Review.

[CR6] Amabile, T. M., & Kramer, S. (2011). *The progress principle: using small wins to ignite joy, engagement, and creativity at work*. Harvard Business Press.

[CR7] Antikainen MJ, Mäkipää M, Ahonen M (2010). Motivating and supporting collaboration in open innovation. European Journal of Innovation Management.

[CR8] Antikainen MJ, Vaataja HK (2010). Rewarding in open innovation communities – how to motivate members. International Journal of Entrepreneurship and Innovation Management.

[CR9] Apergis N, Pekka-Economou V (2010). Incentives and female entrepreneurial activity: evidence from panel firm level data. International Advances in Economic Research.

[CR10] Bandura A (1986). *Social foundations of thought and action: a social cognitive theory*.

[CR11] Bandura A (2001). Social cognitive theory: an agentic perspective. Annual Review of Psychology.

[CR12] Bandura A (2006). Toward a psychology of human agency. Perspectives on Psychological Science.

[CR13] Baranchuk N, Kieschnick R, Moussawi R (2014). Motivating innovation in newly public firms. Journal of Financial Economics.

[CR14] Baregheh A, Rowley J, Sambrook S (2009). Towards a multidisciplinary definition of innovation. Management Decision.

[CR15] Barron KE, Hulleman CS, Eccles JS, Salmelo-Aro K (2015). Expectancy-value-cost model of motivation. *International encyclopedia of social and behavioral sciences*.

[CR16] Bastian, B., Jetten, J., Thai, H. A., & Steffens, N. K. (2018). Shared adversity increases team creativity through fostering supportive interaction. *Frontiers in Psychology*, *9*(Article 2309), 1–10. 10.3389/fpsyg.2018.02309 10.3389/fpsyg.2018.02309 PMC6266683 30532725

[CR17] Bergendahl M, Magnusson M, Björk J (2015). Ideation high performers: a study of motivational factors. Creativity Research Journal.

[CR18] Bessonova E, Gonchar K (2017). Incentives to innovate in response to competition: the role of agency costs. Economic Systems.

[CR19] Bolderdijk, J. W., Brouwer, C., & Cornelissen, G. (2018). When do morally motivated innovators elicit inspiration instead of irritation? *Frontiers in Psychology*, *8*(Article 2362), 1–9. 10.3389/fpsyg.2017.02362 10.3389/fpsyg.2017.02362 PMC5770616 29375454

[CR20] Bowles T, Hattie J (2013). Towards positive adaptive change: the association of three typologies of agency with motivational factors. Australian Psychologist.

[CR21] Carr K, Kendal RL, Flynn EG (2016). Eureka!: What is innovation, how does it develop, and who does it?. Child Development.

[CR22] Chen T, Li F, Leung K (2016). When does supervisor support encourage innovative behavior? Opposite moderating effects of general self-efficacy and internal locus of control. Personnel Psychology.

[CR23] Cordero R, Walsh ST, Kirchhoff BA (2005). Motivating performance in innovative manufacturing plants. Journal of High Technology Management Research.

[CR24] Costa S, Páez D, Sánchez F, Garaigordobil M, Gondim S (2015). Personal factors of creativity: a second order meta-analysis. Journal of Work and Organizational Psychology.

[CR25] Cramond, B. L., & Fairweather, E. C. (2013). Future problem solving as education for innovation. In L. Shavinina (Ed.), *Routledge international handbook of innovation education* (pp. 212–222). London: United Kingdom: Routledge.

[CR26] Curado C, Muñoz-Pascual L, Galende J (2018). Antecedents to innovation performance in SMEs: a mixed methods approach. Journal of Business Research.

[CR27] Curran B, Walsworth S (2014). Can you pay employees to innovate? Evidence from the Canadian private sector. Human Resource Management Journal.

[CR28] De Clercq, D., & Pereira, R. (2019). Resilient employees are creative employees, when the workplace forces them to be. *Creativity and Innovation Management*, (May), 329–342. 10.1111/caim.12328

[CR29] Deci EL, Koestner R, Ryan RM (1999). A meta-analytic review of experiments examining the effects of extrinsic rewards on intrinsic motivation. Psychological Bulletin.

[CR30] Deci EL, Ryan RM (1987). The support of autonomy and the control of behavior. Journal of Personality and Social Psychology.

[CR31] Dee JR, Henkin AB, Pell SWJ (2002). Support for innovation in site-based-managed schools: developing a climate for change. Educational Research Quarterly.

[CR32] Demircioglu MA, Audretsch DB (2017). Conditions for innovation in public sector organizations. Research Policy.

[CR33] Dietrich M, Znotka M, Guthor H, Hilfinger F (2016). Instrumental and non-instrumental factors of social innovation adoption. Voluntas.

[CR34] du Toit ASA, van Staden RJ, Steyn PD (2011). South Africa’s future knowledge workers: a peep into their goals and motivations for innovation. African Journal of Library Archives and Information Science.

[CR35] Duverger P (2012). Variety is the spice of innovation: mediating factors in the service idea generation process. Creativity and Innovation Management.

[CR36] Eccles JS, Wigfield A (2002). Motivational beliefs, values, and goals. Annual Review of Psychology.

[CR37] Ederer F, Manso G (2013). Is pay for performance detrimental to innovation?. Management Science.

[CR38] Edwards RA, Kirwin J, Gonyeau M, Matthews SJ, Lancaster J, DiVall M (2014). A reflective teaching challenge to motivate educational innovation. American Journal of Pharmaceutical Education.

[CR39] Feather, N. T. (1992). Expectancy-Value Theory and unemployment effects. *Journal of Occupational and Organizational Psychology, 65*(4), 315–330. 10.1111/j.2044-8325.1992.tb00508.x

[CR40] Fernandez S, Pitts DW (2011). Understanding employee motivation to innovate: evidence from front line employees in United States federal agencies. Australian Journal of Public Administration.

[CR41] Fischer, C., Malycha, C. P., & Schafmann, E. (2019). The influence of intrinsic motivation and synergistic extrinsic motivators on creativity and innovation. *Frontiers in Psychology*, *10*(Art. 137.). 10.3389/fpsyg.2019.00137. 10.3389/fpsyg.2019.00137 PMC6369195 30778313

[CR42] Flake JK, Barron KE, Hulleman C, McCoach DB, Welsh ME, McCoach BD, Welsh ME (2015). Measuring cost: the forgotten component of expectancy-value theory. Contemporary Educational Psychology.

[CR43] Ford CM (1999). Interpretive style, motivation, ability and context as predictors of executives’ creative performance. Creativity and Innovation Management.

[CR44] Füller J, Matzler K, Hutter K, Hautz J (2012). Consumers’ creative talent: Which characteristics qualify consumers for open innovation projects? An exploration of asymmetrical effects. Creativity and Innovation Management.

[CR45] Galia F (2008). Intrinsic-extrinsic motivations and knowledge sharing in French firms. ICFAI Journal of Knowledge Management.

[CR46] Glassman M (2001). Dewey and Vygotsky: society, experience, and inquiry in educational practice. Educational Researcher.

[CR47] Gopal, G., & College, E. (2011). Using reward systems to motivate employees for innovation. Niall Hoarty, Medtronic Corporation, Galway, Ireland Dr. Gurram Gopal, Elmhurst College, Elmhurst, IL Dr. Laurence P. Elwood, Galway-Mayo Institute of Technology, Galway, Ireland. *Global Education Journal*, 56–67.

[CR48] Gorozidis, G. S., & Papaioannou, A. G. (2014). Teachers’ motivation to participate in training and to implement innovations. *Teaching and Teacher Education, 39*, 1–11. 10.1016/j.tate.2013.12.001.

[CR49] Gorozidis, G. S., & Papaioannou, A. G. (2016). Teachers’ achievement goals and self-determination to engage in work tasks promoting educational innovations. *Learning and Individual Differences, 49*, 46–58. 10.1016/j.lindif.2016.05.014.

[CR50] Green, E. (2013, June 20). Innovation: the history of a buzzword. *The Atlantic.* Retrieved from http://www.theatlantic.com/business/archive/2013/06/innovation-the-history-of-a-buzzword/277067/.

[CR51] Hartmann A (2006). The role of organizational culture in motivating innovative behaviour in construction firms. Construction Innovation.

[CR52] Hopkins V (2016). Institutions, incentives, and policy entrepreneurship. Policy Studies Journal.

[CR53] Horkoff J, Maiden NA, Asboth D (2019). Creative goal modeling for innovative requirements. Information & Software Technology.

[CR54] Hosseini SMP, Narayanan S (2014). Adoption, adaptive innovation, and creative innovation among SMEs in Malaysian manufacturing. Asian Economic Papers.

[CR55] Hsu Y (2009). Exploring design innovation and performance: the roles of issue related to design strategy. Journal of Engineering Design.

[CR56] Jain R, Ali SW (2012). Entrepreneurial and intrapreneurial orientation in Indian enterprises: an empirical study. South Asian Journal of Management.

[CR57] Jean, R.-J. “Bryan”, Kim, D., Chiou, J.-S., & Calantone, R. (2018). Strategic orientations, joint learning, and innovation generation in international customer-supplier relationships. *International Business Review, 27*(4), 838–851. Retrieved from http://10.0.3.248/j.ibusrev.2018.01.007.

[CR58] Jermias J (2007). The effects of corporate governance on the relationship between innovative efforts and performance. European Accounting Review.

[CR59] Jiang M, Thagard P (2014). Creative cognition in social innovation. Creativity Research Journal.

[CR60] Joy S (2004). Innovation motivation: the need to be different. Creativity Research Journal.

[CR61] Kandiko CB (2013). Leadership and creativity in higher education: the role of interdisciplinarity (H05). London Review of Education.

[CR62] Kay L (2011). The effect of inducement prizes on innovation: evidence from the Ansari X Prize and the Northrop Grumman Lunar Lander Challenge. R and D Management.

[CR63] Kinney B, Laux C, Newman P (2015). Executive pay, innovation, and risk-taking. Journal of Economics & Management Strategy.

[CR64] Kirsten B, Du Preez R (2010). Improvisational theatre as team development intervention for climate for work group innovation. SA Journal of Industrial Psychology.

[CR65] Koch AR, Binnewies C, Dormann C (2015). Motivating innovation in schools: school principals’ work engagement as a motivator for schools’ innovation. European Journal of Work and Organizational Psychology.

[CR66] Kruglanski AW, Friedman I, Zeevi G (1971). The effects of extrinsic incentive on some qualitative aspects of task performance. Journal of Personality.

[CR67] Kruglanski AW, Riter A, Amitai A, Margolin B-S, Shabtai L, Zaksh D (1975). Can money enhance intrinsic motivation? A test of the content-consequence hypothesis. Journal of Personality and Social Psychology.

[CR68] Kung, F. Y. H., & Chao, M. M. (2019). The impact of mixed emotions on creativity in negotiation: an interpersonal perspective. *Frontiers in Psychology*, *9*(Article 2660), 1–15. 10.3389/fpsyg.2018.02660 10.3389/fpsyg.2018.02660 PMC6336894 30687150

[CR69] Kuznetsov A, Kuznetsova O (2011). Looking for ways to increase student motivation: internationalisation and value innovation. Higher Education Quarterly.

[CR70] Lam S, Cheng RW, Choy HC (2010). School support and teacher motivation to implement project-based learning. Learning & Instruction.

[CR71] Lehmann-Ortega L, Schoettl J-M (2005). From buzzword to managerial tool: the role of business model in strategic innovation. In *Paper presented at the CLADEA annual assembly*.

[CR72] Lerner J, Wulf J (2018). Innovation and incentives: evidence from corporate R&D. The Review of Economics and Statistics.

[CR73] Liberati A, Altman DG, Tetzlaff J, Mulrow C, Gøtzsche PC, Ioannidis JPA, Moher D (2009). The PRISMA statement for reporting systematic reviews and meta-analyses of studies that evaluate health care interventions: explanation and elaboration. PLoS Medicine.

[CR74] Lopez, S. J., & Snyder, C. R. R. (2003). *Positive psychological assessment: A handbook of models and measures*. *Database.*10.1037/10612-000.

[CR75] Manimala MJ, Jose PD, Thomas KR (2006). Organizational constraints on innovation and intrapreneurship: insights from public sector. Vikalpa: The Journal for Decision Makers.

[CR76] Manso G (2017). Creating incentives for innovation. California Management Review.

[CR77] Maria Stock R, Zacharias NA, Schnellbaecher A (2017). How do strategy and leadership styles jointly affect co-development and its innovation outcomes?. Journal of Product Innovation Management.

[CR78] Marvel MR, Griffin A, Hebda J, Vojak B (2007). Examining the technical corporate entrepreneurs’ motivation: voices from the field. Entrepreneurship: Theory and Practice.

[CR79] Mc Fadden T, Gorman M (2016). Exploring the concept of farm household innovation capacity in relation to farm diversification in policy context. Journal of Rural Studies.

[CR80] Mehta A, Clayton H, Sankar CS (2008). Impact of multi-media case studies on improving intrinsic learning motivation of students. Journal of Educational Technology Systems.

[CR81] Messmann G, Mulder RH (2014). Exploring the role of target specificity in the facilitation of vocational teachers’ innovative work behaviour. Journal of Occupational and Organizational Psychology.

[CR82] Minarcine S, Shaw C (2016). Motivations for entrepreneurship. International Journal of the Academic Business World.

[CR83] Monge, P. R., Cozzens, M. D., & Contractor, N. S. (1992). Communication and motivational predictors of the dynamic of organizational innovation. *Organization Science.*

[CR84] Montani F, Odoardi C, Battistelli A (2014). Individual and contextual determinants of innovative work behaviour: proactive goal generation matters. Journal of Occupational and Organizational Psychology.

[CR85] Mudambi, R., Mudambi, S. M., & Navarra, P. (2007). Global innovation in MNCs: The effects of subsidiary self-determination and teamwork. *Journal of Product Innovation Management, 24*(5), 442–455. 10.1111/j.1540-5885.2007.00262.x

[CR86] Muninger M-I, Hammedi W, Mahr D (2019). The value of social media for innovation: a capability perspective. Journal of Business Research.

[CR87] Naidoo S, Sutherland M (2016). A management dilemma: positioning employees for internal competition versus internal collaboration. Is competition possible?. South African Journal of Business Management.

[CR88] Ng TWH, Feldman DC (2013). Does longer job tenure help or hinder job performance?. Journal of Vocational Behavior.

[CR89] Nold, H. (2017). Using Critical Thinking Teaching Methods to Increase Student Success: An Action Research Project. *International Journal of Teaching and Learning in Higher Education, 29*(1), 17–32. Retrieved from http://www.isetl.org/ijtlhe/.

[CR90] Öberg C, Shih TTY (2014). Divergent and convergent logic of firms: barriers and enablers for development and commercialization of innovations. Industrial Marketing Management.

[CR91] Olivares, S., Saiz, C., & Rivas, S. F. (2013). Encouragement for Thinking Critically. *Electronic Journal of Research in Educational Psychology, 11*(2), 367–394. 10.14204/ejrep.30.12168.

[CR92] Ozorhon B, Oral K (2017). Drivers of innovation in construction projects. J. Constr. Eng. Manage..

[CR93] Phillips R (2004). The global export of risk: finance and the film business. Competition & Change.

[CR94] Pihie ZAL (2007). An analysis of academic experience to develop entrepreneurial attributes and motivation among at-risk students. The International Journal of Learning.

[CR95] Pihlajamaa M (2017). Going the extra mile: managing individual motivation in radical innovation development. Journal of Engineering and Technology Management.

[CR96] Piperopoulos P, Wu J, Wang C (2018). Outward FDI, location choices and innovation performance of emerging market enterprises. Research Policy.

[CR97] Poskela J, Martinsuo M (2009). Management control and strategic renewal in the front end of innovation. Journal of Product Innovation Management.

[CR98] Radicic, D., Pugh, G., Hollanders, H., Wintjes, R., & Fairburn, J. (2016). The impact of innovation support programs on small and medium enterprises innovation in traditional manufacturing industries: an evaluation for seven European Union regions. *Environment & Planning C: Government & Policy*, *34*(8), 1425–1452. Retrieved from 10.0.4.153/0263774X15621759

[CR99] Reznickova A, Zepeda L (2016). Can self-determination theory explain the self-perpetuation of social innovations? A case study of slow food at the University of Wisconsin-Madison. Journal of Community & Applied Social Psychology.

[CR100] Romero, I., & Martínez-Román, J. A. (2012). Self-employment and innovation. Exploring the determinants of innovative behavior in small businesses. *Research Policy, 41*(1), 178–189. Retrieved from http://10.0.3.248/j.respol.2011.07.005.

[CR101] Ryan RM, Deci EL (2017). *Self-determination theory: basic psychological needs in motivation, development, and wellness*.

[CR102] Scott SG, Bruce RA (1994). Determinants of innovative behavior: a path model of individual innovation in the workplace. The Academy of Management Journal.

[CR103] Senko C, Hulleman CS, Harackiewicz JM (2011). Achievement goal theory at the crossroads: old controversies, current challenges, and new directions. Educational Psychologist.

[CR104] Sergeeva N, Zanello C (2018). Championing and promoting innovation in UK megaprojects. International Journal of Project Management.

[CR105] Shane, S., Locke, E. A., & Collins, C. J. (2003). Entrepreneurial motivation. *Human Resource Management Review*, *13*(2), 257–279. 10.1016/S1053-4822(03)00017-2

[CR106] Skinner NF (1996). Behavioral Implications of Public Service Motivation. Social Behavior and Personality.

[CR107] Skinner NF, Drake JM (2003). Behavioral implications of adaption-innovation: III. adaption-innovation, achievement motivation, and academic performance. Social Behavior and Personality: An International Journal.

[CR108] Smith G, Sandberg J (2018). Barriers to innovating with open government data: exploring experiences across service phases and user types. Information Polity: The International Journal of Government & Democracy in the Information Age.

[CR109] Soleas, Eleftherios K. (2020). Expectancies, values, and costs of innovating identified by Canadian innovators : a motivational basis for supporting innovation talent development. *Journal of Advanced Academics*, 1–25. 10.1177/1932202X20904772.

[CR110] Soleas, Eleftherios Kyprianos. (2018a). True “innovation” generates ideas, not wealth. *The Conversation*. Retrieved from https://theconversation.com/amp/true-innovation-generates-ideas-not-wealth-103590.

[CR111] Soleas EK (2018). Get off my lawn: why capitalism’s monopoly on innovation is bad for us all and what educators can do about it. Graduate Student Symposium Selected Papers.

[CR112] Song M, Im S, Van Der Bij H, Song LZ (2011). Does strategic planning enhance or impede innovation and firm performance?. Journal of Product Innovation Management.

[CR113] Sorice MG, Donlan CJ (2015). A human-centered framework for innovation in conservation incentive programs. Ambio.

[CR114] Spanjol J, Tam L (2010). To change or not to change: how regulatory focus affects change in dyadic decision-making. Creativity and Innovation Management.

[CR115] Stewart WH, Watson WE, Carland JC, Carland JW (1999). A proclivity for entrepreneurship: a comparison of entrepreneurs, small business owners, and corporate managers. Journal of Business Venturing.

[CR116] Susha I, Grönlund A, Janssen M (2015). Driving factors of service innovation using open government data: an exploratory study of entrepreneurs in two countries. Information Polity.

[CR117] Thapa BEP, Niehaves B, Seidel CE, Plattfaut R (2015). Citizen involvement in public sector innovation: government and citizen perspectives. Information Polity.

[CR118] The Campbell Collaboration (2017). Campbell systematic reviews: policies and guidelines. Campbell Policies and Guidelines Series No. 1.

[CR119] Vansteenkiste M, Lens W, Witte H, Feather NT (2005). Understanding unemployed people’s job search behaviour, unemployment experience and well-being: a comparison of expectancy-value theory and self-determination theory. British Journal of Social Psychology.

[CR120] Vygotsky LS, Kozulin A (2011). The dynamics of the schoolchild’s mental development in relation to teaching and learning. Journal of Cognitive Education and Psychology.

[CR121] Wang CKJ, Liu WC, Koh C, Tan OS, Ee J (2011). A motivational analysis of project work in Singapore using self-determination theory. The International Journal of Research and Review.

[CR122] Wang G, Huang H (2015). Effect of Chinese employees’ emotional creativity on their innovative performance. Social Behavior and Personality.

[CR123] Wang, Y., Liu, J., Zhu, Y., & Bastian, B. (2018). Humble leadership, psychological safety, knowledge sharing, and follower creativity: a cross-level investigation. *Frontiers in Psychology*, *9*(Article 1727), 1–9. 10.3389/fpsyg.2018.01727. 10.3389/fpsyg.2018.01727 PMC6156152 30283379

[CR124] Wendelken A, Danzinger F, Möslein K, Rau C (2014). Innovation without me: why employees do (not) participate in organizational innovation communities. R&D Management.

[CR125] Wigfield A (1994). Expectancy-value theory of achievement motivation: a developmental perspective. Educational Psychology Review.

[CR126] Wigfield A, Tonks S, Klauda SL, Wentzel KR, Wigfield A (2009). Expectancy-value theory. *Handbook of motivation at school*.

[CR127] Wu A, Su J, Wang H (2013). Internal innovation or external innovation? An organizational context-based analysis in China. Journal of High Technology Management Research.

[CR128] Wu HL, Su WC, Lee CY (2008). Employee ownership motivation and individual risk-taking behaviour: a cross-level analysis of Taiwan’s privatized enterprises. International Journal of Human Resource Management.

[CR129] Xie Y, Reider D (2014). Integration of innovative technologies for enhancing students’ motivation for science learning and career. Journal of Science Education and Technology.

[CR130] Yidong T, Xinxin L (2013). How ethical leadership influence employees’ innovative work behavior: a perspective of intrinsic motivation. Journal of Business Ethics.

[CR131] Zheng H, Li D, Hou W (2011). Task design, motivation, and participation in crowdsourcing contests. International Journal of Electronic Commerce.

